# Error in Text

**DOI:** 10.1001/jamanetworkopen.2019.14712

**Published:** 2019-10-09

In the Editorial titled “Physical Activity, Fitness, and Cardiovascular Health: Insights From Publications in *JAMA Network Open*,”^1^ published August 23, 2019, there was an error in the wording of the fourth sentence of the eighth paragraph. This sentence should have read, “The authors observed associations of aerobic fitness with metabolome markers of cardiovascular health that were largely attenuated after accounting for body fat percentage.” This article has been corrected.^1^

## References

[1] Bradley SM, Michos ED, Miedema MD Physical activity, fitness, and cardiovascular health: insights from publications in *JAMA Network Open.* JAMA Netw Open. 2019;2(8):. doi:10.1001/jamanetworkopen.2019.8343 31441931

